# Good Old-Fashioned Artificial Consciousness and the Intermediate Level Fallacy

**DOI:** 10.3389/frobt.2018.00039

**Published:** 2018-04-18

**Authors:** Riccardo Manzotti, Antonio Chella

**Affiliations:** ^1^Department of Business, Law, Economics and Consumer Behavior, Università di Comunicazione e Lingue (IULM), Milan, Italy; ^2^RoboticsLab, Department of Industrial and Digital Innovation, University of Palermo, Palermo, Italy; ^3^Cognitive Robotics and Social Sensing Laboratory, ICAR-CNR, Palermo, Italy

**Keywords:** robot consciousness, machine consciousness, artificial consciousness, synthetic phenomenology, robot self-awareness

## Abstract

Recently, there has been considerable interest and effort to the possibility to design and implement conscious robots, i.e., the chance that robots may have subjective experiences. Typical approaches as the global workspace, information integration, enaction, cognitive mechanisms, embodiment, i.e., the Good Old-Fashioned Artificial Consciousness, henceforth, GOFAC, share the same conceptual framework. In this paper, we discuss GOFAC's basic tenets and their implication for AI and Robotics. In particular, we point out the intermediate level fallacy as the central issue affecting GOFAC. Finally, we outline a possible alternative conceptual framework toward robot consciousness.

## Introduction

Consciousness exists: we are conscious, and it would be odd to negate this fact. Consciousness is a part of our physical world, and then the processes at the basis of consciousness must be faced by the laws of science governing our physical world.

The definition of consciousness is still an open question. Therefore, it would be problematic to discuss about robot consciousness: in facts, Raoult and Yampolskiy (2015) reviewed 21 proposed tests presented in the literature to assess consciousness in machines and robots. However, the same situation holds for other complex concepts: notably, Legg and Hutter (2007) review more than 70 existing different definitions of “intelligence.” The fact that there is no agreement on what intelligence is does not refrain researchers to speaking about Artificial Intelligence.

In facts, consciousness is an important research topic in neuroscience: Dehaene (2014) summarizes several years of studies in human consciousness; see also Tononi (2012) and Damasio (2010), among others. Notably, neuroscientists working on consciousness take seriously into account the possibility that, in the near future, robots may be conscious. During the Symposium organized in 2001 by the Swartz Foundation on “Can a Machine Be Conscious,” the concluding remarks of Christof Koch stated that:

“we know of no fundamental law or principle operating in this universe that forbids the existence of subjective feelings in artifacts designed or evolved by humans.^1^”

To the best of our knowledge, this claim is valid still today.

Consciousness is part of our physical world, and then some of its aspects may be studied and even replicated by using robots. On the one hand, the employment of robots as tools may help to understand biological consciousness better, and, on the other hand, the processes at the basis of consciousness may be in some sense crudely replicated to build better robots, as it happened, e.g., for neural networks and artificial life systems. Anil Seth has claimed that:

“Over the last *two* decades much has changed […]. Alongside philosophical discourse a new science of consciousness has taken shape which integrates experimental and theoretical work cross many fields including neuroscience, psychology, cognitive science, artificial intelligence, computer science, neurology, and psychiatry.” (Seth, 2010, p. 1).

It is not a case that the late Nobel prize Gerald Edelman, a scholar of the research on consciousness, employed robots to validate parts of his theory of consciousness (Reeke et al., 1990; Edelman et al., 1992). Koch and Tononi directly addressed the possibility that artifacts may be conscious by taking into account constraints and conditions according to the Integrated Information Theory of consciousness (see below). Notably, Koch and Tononi (2008, 2017) explicitly discussed and proposed tests for consciousness in the machines. Recently, Dehaene et al. (2017) summarized the neuroscientific findings of interest for conscious machines. We concur with their claim according to which the study of biological consciousness may inspire novel machine architectures.

In this direction, the paper by Grossberg (2017) summarizes years of works about brain resonances and proposes a set of models, described by differential equations, that captures some of the main aspects of consciousness. Important papers in this line are due to Perlovsky (2006, 2016) where he claims that a new “physics of mind” is needed that looks for the fundamental laws of the material world, including sentience. The new physics of mind should develop the mathematical theories that explain the empirical evidence about sentience and that generate suitable predictions to be verified by experiments.

Therefore, the problem of consciousness in robots and artifacts is an accepted issue for researchers in neuroscience.

In the AI debate, the problem of machine consciousness has been discussed by many scholars since the seminal paper by McCarthy (2002), where he considered an extension of the Situation Calculus to deal with some aspects of self-reflection to make robot conscious of their mental states. On a similar line, McDermott (2001) devoted his book on “Mind and Mechanisms” on the discussion of a computational theory of consciousness.

Many journals and conference papers discussed the possibility of consciousness in machines and robots by proposing theories and architectures. Holland (2003) and Chella and Manzotti (2007) collected the initial attempts at robot consciousness. An almost complete up to date review is due to Reggia (2013). Scheutz (2014) reviewed and discussed the contact points between machine consciousness and artificial emotions.

Among the essential works from AI scholars concerning machine and robot consciousness, we mention, among others, the architectures based on the global workspace model of consciousness (Baars, 1997) as the LIDA architecture (Franklin, 2003; Franklin et al., 2014) and the cognitive architecture proposed by Shanahan (2005, 2006). A model of conscious experience related to learning and sensorimotor interaction in an autonomous robot has been discussed by Kuipers (2008). Notably, Bringsjord et al. (2015) recently implemented a cognitive system based on higher-order logic running on the NAO robot that passed human tests of self-consciousness.

Therefore, robot consciousness is an important research field that benefits from the contributions of many scholars from neuroscience, Artificial Intelligence, and robotics. The general feeling is, as stated above, that understanding biological consciousness may help to build better robots and, on the other side, that the research on robot consciousness may help understanding biological consciousness.

This paper aims to propose a critical review and analysis of the literature related to robots and machine consciousness under the light of what we named the “intermediate level fallacy.” In facts, many theories of machine consciousness actually do not directly address the problem of consciousness, but they discuss some intermediate problem, then leaving aside the issue of robot consciousness.

Then, the goal of the paper is not to discuss a specific algorithm or software, but to help roboticists interested in robot consciousness to build a mental map of the bibliography in the field and to avoid quirks due to the intermediate level fallacy.

## Good old fashioned machine consciousness

As previously stated, in recent years the notion of machine and robot consciousness gained momentum and attracted considerable interest. Chella and Manzotti (2009) discussed many problems arising in the assessment of consciousness in a robot concerning the role of the body, the needs for the robot to be “situated” in an environment, the cognitive capabilities of the robot, the effective functions of emotions and so on.

The most challenging problem for robot consciousness is the possibility that a robot may have real subjective experiences. However, many approaches at the state of the art in robot consciousness are biased by a set of premises that harnessed research into what can be named as Good Old-Fashioned Artificial Consciousness (GOFAC).

GOFAC suggests a physical world in which consciousness appears as a result of a specific intermediate level. A theory based on the idea that consciousness emerges from an intermediate level should explain what this level is and why it produces consciousness. However, the explanation is problematic because, rather than explaining consciousness, the theory introduces a new level as an intermediate entity, that is only apparently less troublesome. In contrast, the intermediate level is explanatory disruptive since it adds two new problems: the characteristics of the new level and its relation to consciousness. This approach can be named the “intermediate level fallacy” and it seems to be attractive because the introduced level appears less intimidating and more familiar than consciousness itself.

This paper aims to list some of the leading approaches to robot consciousness under the light of the intermediate level fallacy. While each method has its peculiar shortcomings, they share the standard pattern, i.e., the intermediate level fallacy, that characterizes GOFAC. They try to downgrade the notion of consciousness to something more amenable—a move that does not solve, but it multiplies the problems. After analyzing a series of well-known approaches, the paper outlines a possible direction in which research might go to overcome GOFAC.

This paper has not a negative goal, namely to list a series of hypotheses and premises and stress the overall failure of GOFAC. Instead, it aims to shed light on some, likely fruitful direction of research.

## The hard problem

The main culprit behind GOFAC is David Chalmers's introduction of the *hard problem* (Chalmers, 1996). According to Chalmers's seminal book, most of research and discussion about consciousness has been carried on inside the conceptual framework set by the contrast between a *conscious* mind and a *cognitive* mind. Such a notion has entrenched the gap between subjective, *phenomenal* experience and *physical* properties. The hard problem—namely the idea that once all the material facts are fixed, there is still something to be explained, has postponed the understanding of consciousness and placed it outside of robot implementation. If one accepts it, it follows that a robot will never be genuinely conscious because no matter how all physical facts are fixed, there will still be something to be added. The acceptance of the hard problem is the main reason behind the ensuing lack of progress in robot consciousness.

The hard problem is based on the premise that subjective and physical properties are alien to each other. Moreover, yet, this premise is not of experimental nature, and it might be questioned. In facts, if subjective and physical properties are different, then it would be impossible to place them against each. Consider, for example, the comparison between *subjective red* and *real red*. There is no reason to believe there are two kinds of red. Of course, the usual claim is that the subjective red is of mental nature and the physical world is not accessible in a profound sense. Chalmers claims that there are only subjective properties, or, to use an equivalent and famous formulation, that we only experience the phenomenal character of what happens.

This claim is unsupported by the facts that human beings experience the external world and their own body. It is not phenomenal; it is just what the physical world is. There are no reasons to assume, as Chalmers does, that the perception of the world is different from the physical world. There are no perceptions of subjective properties, but instead, human beings experience the attributes the world is made of, and the name we can give to such characteristics is physical.

The hard problem is not empirically grounded because if it were true, it could not be empirically proven. If consciousness were hard, it could not affect the physical world. Conversely, if consciousness were testable, it would not be hard.

The hard problem is related to the *epiphenomenal* conception of consciousness, i.e., that consciousness has no physical role. Accepting the hard problem means that consciousness will be external to the domain of material facts. In fact, if consciousness were part of the physical world, it could be measured, observed, replicated, designed and implemented in a robot. The hard problem encourages to conceive of consciousness as something intractable by scientific means. Consciousness could not have any effect on the physical world and, consequently, it would be useless from a robotics perspective.

However, if consciousness is epiphenomenal, it would contradict the selective advantage that it seems to provide. Moreover, there are no other natural phenomena that are deemed to be epiphenomenal. All physical events are causally relevant, that is why they can be measured and observed them, as they exert a causal effect. In physics and engineering, there are no such phenomena because they would be automatically deemed not to be real. The fact that GOFAC deals with consciousness as epiphenomenal is the hallmark of scientific failure. Once inside the traditional GOFAC framework, then consciousness is outside of empirical reach.

The notion of epiphenomenal consciousness appears to be a self-defeating hypothesis. Human beings as conscious agents have a feeling that what they feel is interwoven with the physical world. Consciousness is indeed a part of the physical world, and if the current scientific picture of the world does not have a place for consciousness, then it is not complete.

Nonetheless, the hard problem became famous also because it contrasts the *easy* problems—how to explain the human ability to recognize a face, generate language, control behavior—from the hard problem of defining how physical processes can give rise to consciousness. Such a split suggests, on the one side, that scientists could continue their work without worrying about consciousness and, on the other hand, that consciousness is elusive and not constrained by the physical world. It also provides engineers, roboticists, and AI experts free to design robot consciousness as long as they were smart enough to leave the hard problem aside and limit themselves to the easy problems of consciousness.

In GOFAC, the hard problem spawned a split between hard and weak machine consciousness (Seth, 2009) as though it were possible to focus on functional and ontological problems separately. Because of the widespread acceptance of the hard problem, scholars assumed that conscious experience is out of reach of science and technology and thus that a workaround has to be proposed. The workaround was the delusion that it is possible to focus on concrete problems—i.e., those that are part of our conceptual framework—and to leave the real issue of consciousness to some conceptual breakthrough.

The above state of things suggests that the literature on robot consciousness does not deal with phenomenal consciousness. Consciousness has been dropped from the physical world by the hard problem, and thus it has been become legitimate to study it without addressing the crux of the matter.

## The intermediate level fallacy

Given the starting conceptual landscape shaped by the acceptance of the hard problem—or some version of it—a widespread tendency has been that of looking for some workaround. A common strategy has been that of the intermediate level which is composed of two steps. First, an intermediate conceptual level that is at a possible explanatory distance is proposed—behavior, central workspace, information, enaction, adaptive resonance, and so forth. Such an entity, crucially, is located on the physical side of the gap but, equally significantly, it is somewhat vague, to the extent that it may suggest some degrees of consciousness. Second, consciousness is watered down to show that it is not much better than the intermediate level. The second step, which is most problematic from an ontological and epistemic perspective, is critical to provide fulfillment of the first step.

As an example of the intermediate level fallacy, consider Seth's proposal to look for a real problem rather than for the hard or the easy problem. According to Seth, the real question consists in examining

“how to account for the various properties of consciousness regarding biological mechanisms; without pretending it doesn't exist (easy problem) and without worrying too much about explaining its existence in the first place (hard problem).” (Seth, 2016).

The real problem, according to Seth, is nothing but one of the traditional easy problems in disguise. In this case, the intermediate level is represented by the biological mechanisms that are physical processes that do not qualify as a solution to the hard problem. In this regard, Seth himself defended weak machine consciousness (Seth, 2009). So, it is not clear why the real problem according to Seth should be a successful research strategy for consciousness. It is the second step of the intermediate level strategy, i.e., watering down consciousness. Seth's catchphrase is that

“It looks like scientists and philosophers might have made consciousness far more mysterious than it needs to be” (Seth, 2016).

Thus, he suggests that, after all, there is no mystery. In fact, Seth argues that

“In the same way, tackling the real problem of consciousness depends on distinguishing different aspects of consciousness, and mapping their phenomenological properties (subjective first-person descriptions of what conscious experiences are like) onto underlying biological mechanisms (objective third-person descriptions)” (Seth, 2016).

In his account, the problem of consciousness is no longer that of tackling an apparently impossible feat for the physical world, but a mapping between personal reports onto biological mechanisms. This mapping may be tedious but feasible. However, such a mapping does not offer a solution of the problem of consciousness. Both personal descriptions and biological mechanisms are objective physical phenomena that pose no threat to the received view of physics. Both of them do not address the issue of consciousness.

Then, the first step of the fallacy is to suggest an intermediate, safe level of explanation, like a suitable biological mechanisms. The second step is to water down the problem of consciousness to something more amenable as the mapping between personal reports and the biological mechanisms.

## Current approaches to robot consciousness

Robot consciousness has so far not succeeded in making progress on the issue of phenomenal experience. While the possibility of conscious machines, together with its ethical implications, has repeatedly been addressed, no one has claimed that anything close to a feeling has occurred in an artifact. As before, this persistent and generalized lack of results might be explained by the adoption of the familiar and flawed conceptual landscape of GOFAC. In particular, the intermediate level fallacy is a common problem in all these attempts. Here, we will consider, as possible theoretical backgrounds for machine consciousness, functionalism, information, embodiment, enaction and cognition. We will argue that these approaches exhibit the manifest symptoms of the fallacy and are as many cases of GOFAC.

### Functionalism

Functionalism is the backbone of the AI approach to consciousness. Functionalist approaches single out a functional view of the mind. This critique has been developed at length by many scholars, most notably Searle (1990) and Harnad (2003). If the mind is a collection of functional relations, no space is left for what is taken to be consciousness—functioning vs. feeling, to use Harnad's formulation. Functionalism focuses on external causal relations between the state of affairs. While functionalism is neutral to the location of such causal relationships, it concentrates mostly on abstract descriptions of reality, which is the reason why it allows multiple realizations. Functionalism is a theoretical description of what goes on in a system, and it is oblivious to the physical constituents of a system. Therefore, functionalism will never grasp consciousness because it is neutral to the material components of functional relations.

Then, functionalism would provide the same description for a system made of neurons and of electronic switches, and it will offer the same explanation for a system with consciousness and without consciousness. It is not a fact about consciousness; it is a consequence of the premises on which functionalism is built.

Functionalism has been ideal to back up the philosophical notion of a *zombie*, which was fundamental in all the accounts inspired by the hard problem (Chalmers, 1996). A zombie is an entity which externally is not distinguishable from a human being, in the sense that it talks, it responds, it acts in the world, but, contrary to a human being, it is entirely unconscious. The conceivability of a zombie tells us more about the limitation of functionalism than about consciousness. There is no evidence that a physical entity identical to a human being might be without consciousness. The notion of a zombie shows that functional descriptions are incomplete and leave out something crucial. In fact, in practice, all machines nowadays are considered philosophical zombies. No one expects Siri or Google Assistant to be anything but zombies.

Many approaches to consciousness are functionalist models. Consider the mentioned global workspace model (Baars, 1997) and its implementations (Shanahan, 2005, 2006; Franklin et al., 2014). Such a model is constituted by a suitable functional structure where the information is lumped and broadcasted. The first step is represented by the particular cognitive structure, the central workspace, that is a neutral concept, and that takes into account the notion of unity and the idea of a central controller. The second step is the watering down of consciousness, namely the claim that, to be conscious is nothing but accessing information in a centralized fashion.

Another approach is the model of consciousness formulated by Stephen Grossberg (2007, 2017) and based on adaptive resonances in the brain. According to this model, the conscious states in the brain are characterized as resonant neural states, i.e., neural states where the firing of neurons are mutually amplified and synchronized thanks to feed-forward and feedback connections between bottom-up and top-down neural layers. In this case, the first step of the move is represented by a suitable characteristic of the dynamic evolution of a neural network, i.e., the resonance of interconnected neurons, which is a neutral effect that is explained by the differential equations governing the dynamics of neural networks. The second step is the claim that subjective experience is nothing but this particular state in the dynamic evolution of neural networks. Of course, not any rationale has been presented as to why centralized accessed information or a resonant state could not be unconscious. The presence of the fallacy is evident.

It is not to say that robots envisaged by functionalist designers will never be conscious. In fact, designers, no matter what conceptual frameworks they employ, when they move from designing to implementations, are subject to the structure of the physical world. Thus, their products are not limited by their conceptual models. As consciousness is part of the natural manifold, there will be cases in which the physical structure of agents will yield to consciousness, no matter the conceptual framework adopted by its designers.

### Information and computation

Another popular approach in GOFAC is based on seeking unique information processes that produce consciousness. Information, at the level of computational processes such as those implemented by brains or by computers, is not a physical constituent of reality. Instead, it is a convenient level of description. Information is a fictitious entity, like a center of mass or a meridian: it is not physically there, but it exists only in our descriptions. It cannot be observed, but, significantly, calculated.

In the case of information, there is confusion among scientists. The everyday familiarity with information has fostered a widespread tendency to deal with information as though it were real, like water or electricity. However, there is no evidence that information is anything over and above the physical processes we describe using an informational jargon (Shannon, 1948; Searle, 1984); it is nothing but a quantitative description of the causal relations between events. From a physical perspective, there is no need for an additional level called information over and above the physical phenomena, but all the causal power is drained by physical events (Kim, 1989, 1998; Dowe, 2000, 2007).

As an argument of the fact that information does not have a physical existence consider that if information were real, it should be possible to build an information detector. Interestingly, it is not possible to construct an information detector. While it is possible to compute the amount of information inside a system from a set of assumption as to how that system is going to be exploited, it is impossible to detect the amount of information in a system. For instance, if one knows that a CD-Rom is going to be read by a standard CD-Player one can compute its capacity. However, if one takes a piece of matter and one does not know whether and how its physical structure is going to be exploited, one cannot know how much information it contains. The same holds in all cases of similar information devices. It is not possible to measure information as say, mass, electric charge, length. Information can be *estimated* or *computed* based on what it is known about a piece of matter and its role in a given context.

In sum, information does not exist except as a way to describe what does happen between causally coupled events, coherently with the original formulation of information (Shannon, 1948). Information is a way to explain causal processes; it is not a real phenomenon. It is not physical insofar it is causally redundant, undetectable, never measured but only estimated. On top of that, there would be no law explaining why a specific informational state should be like a conscious state.

Information-based approaches to consciousness remain in the intermediate level fallacy. The intermediate entity is now information—sometimes a specific brand of information as in Tononi's integrated information theory (Tononi, 2004) and its most recent version (Oizumi et al., 2014). The watering down is the effort to claim that the properties of information are those that matter for consciousness. For instance, Tononi claimed that integrated information has unity and that consciousness too has unity. Concerning quality, semantics, content, and all other aspects of our experience, he does not have any word.

In sum, approaches like those suggested by Tononi and based on the idea that information processing produces consciousness, are empirically not founded because information has not a physical reality. They are biased by the hope that a quantitative, precise method may offer a scientific framework. In fact, these authors emphasize the possibility to *measure* consciousness. At most, these methods can succeed in estimating informational states that correlate with consciousness, but, so far, they have been unable to present justification as to why the informational states under scrutiny should constitute consciousness.

### Embodiment

In robot consciousness, popular approaches are related with the notion of embodiment (Holland, 2004; Bongard et al., 2006; Shanahan, 2006, 2010) mostly because they allow focusing on robot bodies. It is a fruitful approach that highlights crucial features of the embodiment. The body plays an essential role in shaping the interaction between an agent and its environment. Embodied cognition is a mandatory perspective regarding sensory-motor loops. However, it is not clear why embodiment should provide clues on how consciousness fits with the physical world. Inevitably, embodiment simplifies many critical sensory-motor control loops.

If embodiment refers to the fact that a cognitive or conscious process must be physically embodied, it is a pretty obvious notion. A cognitive process must be embodied in this sense, as any process must correspond to something physical and thus be embodied. However, supporters of the concept of embodiment as Chrisley and Ziemke (2006) mean something less trivial.

These authors compete against the traditional notion of cognition as a higher order process carried on by a central processing unit physically separate from the body. Such an approach is the offshoot of historical factors—i.e., mostly, the Cartesian notion of an immaterial mind, a functionalist model of the mind, and the availability of electronic calculators well before they could be coupled with artificial bodies. All these factors fostered a disembodied notion of the mind and its processes. However, they have long ceased to be relevant, both in the philosophical debate as well as in the technological playground.

AI is biased by a Cartesian view of the mind. Embodiment allowed AI scholars to emphasize the physical nature of agent hood. However, this fact does not imply that the body is the only constituent of an agent.

The notion of embodiment self-contradicts its original intentions. In fact, the embodiment was taken into consideration to get rid of the immaterial mind, as the body and its interaction with the world appear like a feasible solution. Unfortunately, the notion of “body” is unclear. Typically, an object is a body only when it is the body of a subject. However, then, the notion of the body is circularly the cornerstone of the subject. The body is another intermediate entity that should bridge the gap between world and consciousness. It is the symptom of the intermediate level fallacy. The body—or its interactions with the environment—is proposed as the intermediate level. At the same time, the watering down step deals with the body as though it were something more than a moving physical object. The last step is, of course, of relevance in the case of robot consciousness where researchers do not have a biological body. The features that should be present in an object to be qualified as a body are not explained. In this sense, a washing machine may be considered as a body, because it reacts to external stimuli, it swallows stuff, it processes it, it expels it, it consumes energy, it plans. The same arguments hold for anthropomorphic robots (Holland, 2003; Natale et al., 2012).

Thus, embodiment tries to exploit the intermediate level fallacy by employing the ambiguous notion of a body, and to water down consciousness to something more mundane as the body.

### Enaction

Another viable solution to achieve robot consciousness is offered by enaction insofar as it suggests that experience is constituted by a body and its interactions and with the world, and thus it may be implemented in artifacts (O'Regan and Nöe, 2001).

Enactivism defends a firm stance that, together with the embodiment is likely to be productive in many fields, most notably cognitive science (Stewart et al., 2010). What enaction has never addressed is the enactive level of reality and why there should be anything like that—namely the first step of the fallacy.

Consider the basic tenet of enaction, in Alva Noë's formulation:

“Perceiving is a way of acting […] What we perceive is determined by what we are ready to do […] We enact out perception; we act it out” (Noë, 2004, p. 1).

Once again, Noë suggests an intermediate level based on actions, that should underpin perception. Of course, he does not explain why actions should be different in the case they are performed by human bodies from the case in which they are performed by a robot or an animal.

Enactivism does not provide a criterion to distinguish between real actions and simple movements unless by reference to subjects. In other words, an act is a movement performed by a subject with intentions and understanding—i.e., a conscious subject. Then there is the concrete risk of circularity in their arguments. Consider this point in John Stewart's formulation:

“How can a material state *be* a mental state? Hoary it may be, yet the problem is anything but solved. […] The paradigm of enaction solves this problem by grounding all cognition as an essential feature of living organism” (Stewart, 2010, p. 1).

Of course, as Stewart himself admits, this does not solve the problem. It only shifts the burden of the explanation on the notion of the living organism. Since vitalism has long been dismissed, the emphasis on life and living organisms does not seem a convincing conceptual fulcrum. In this way, the suggested intermediate level is the living organism and its feedback loops with the external world. Why these phenomena should be any special is left unexplained. It is the second step of the fallacy.

Finally, it is characteristic of enaction the shift from actions as such to knowledge about actions. In fact, recent accounts of consciousness in enaction take stock of the notion of knowledge. In this regard, Noë claims that

“To be a perceiver is to understand, implicitly, the effects of movement on sensory stimulation.” (Noë, 2004, p. 1).

Once again, an intermediate level, that of understanding and sensory-motor knowledge, is presented as a way to reach consciousness. What such an intermediate level is in a physical world and why knowledge of the effects of movement on sensory stimulation should lead to conscious experience is not clear at all.

### Cognition and intelligence

The most obvious candidate for consciousness is cognition and intelligence. Here, we have a promising intermediate entity which looks apparently less demanding, and we may consider whether it might be the right ladder. After all, there seems to be a tight connection between cognitive capabilities and consciousness. Most of the time, when a human being exerts higher-order cognitive processes are conscious. However, it is fair to maintain that, in many cases, when one is conscious very little intelligence is required or that many of the most creative ideas have been the outcome of mostly unconscious activities (Lavazza and Manzotti, 2013).

It is a fact that many scholars are tempted to focus on intelligence and cognition and expect that consciousness will come for free once all the practical issues have been solved. Alternatively, instead many hold that the problem will evaporate as a false problem.

However, also, in this case, cognition is an intermediate level that may lead to the knowledge of consciousness, and not to consciousness experience. Also, this is a symptom of the intermediate level fallacy.

## What is left?

We found a common explanatory strategy in the reviewed attempts. Scholars working in robot consciousness suggest an intermediate level—sensory-motor patterns, information, cognition, global workspace—as a possible explanation for consciousness. What is missing is why such a level should lead to consciousness. From an epistemic perspective, it is as though they suggested an *explanans* without providing its relationship with the *explanandum*, i.e., consciousness. Table 1 summarizes the different GOFAC landscapes of the intermediate level fallacy.

**Table 1:** The intermediate level fallacy in different GOFAC landscapes.

	**Actual physical world**	**Intermediate level**	**Watered down version of consciousness**
Functionalism	The physical states that realize functional structures	Global workspace, centralized representations, adaptive resonance	Access consciousness
Information and computation	The physical states that transmit causal processes	Integrated Information	Integrated consciousness
Embodiment	Objects	Body states, body-world states	Sensory-motor loops
Enaction	Interactions between objects and environment	Actions	Knowledge of sensory-motor loops
Cognition	Brain or processor	Cognitive states	Knowledge

The hard problem, the GOFAC approaches, and the strong vs. weak machine consciousness argument are all grouped by a common factor, as they all deal with consciousness as lacking any causal role in the world. Consider for example the hard problem, that leads to the issue of the zombie, a cognitively equivalent agent lacking consciousness. In turn, GOFAC does not address the issue of subjective experience. Finally, the split between weak and strong machine consciousness was conceived to deal with cognitive processing without addressing the crux of the matter, namely conscious experience. Weak consciousness was designed to deal with the functional aspects of consciousness—i.e., those with causal relevance—and therefore to leave out strong consciousness.

New hypotheses about the nature of the physical world are needed. Consciousness is a fact that needs to find its place in nature. Thus, if consciousness is neither of the previously examined processes what is left? The proposal is that consciousness is the structure of the physical world itself. Such a move has been except in some cases, as in Perlovsky (2006, 2016). There must be fundamental mistakes in the way the physical world is conceived. A possible error might be the location of the thing called consciousness in a different place rather than the body of the agent or the neural/computational structure. Another mistake might consist in the split between the subject and the object. The paper shows that GOFAC will never achieve machine consciousness and thus that it clamors for the adoption of a robust conceptual framework alternative to the hard problem and its cognates.

Of course, finding consciousness inside the physical world is necessary when the goal is designing a conscious robot. A robot does not have any other resource but those offered by the physical world. It may sound like a platitude but, give or take, all mentioned approaches run according to this principle. Therefore, any viable solutions will require setting aside the premise that has so far hampered any progress—i.e., the hard problem with the general belief that consciousness is something distinct from the physical world. We have to reconsider the question from the beginning.

We believe it is possible to flesh out a radical alternative that will stem from setting aside the obnoxious theoretical framework fostered by the adoption of the Hard Problem. First, we take consciousness to be just like all other physical properties around, something that can be measured, observed. Furthermore, consciousness is causally active and located in space-time. Finally, it is made of matter or energy. These premises are nothing more than restating the assumption that consciousness is physical. In fact, everything that is physical is spatiotemporally located, causally relevant, made of matter/energy, and observable. So much the worse for epiphenomenalism and zombies.

Of course, this move will be considered unfeasible by most scholars insofar as they take consciousness to be invisible in the physical world. Neuroscientists have been looking for it inside the brain for the last couple of centuries without finding anything resembling it. In the brain, there is nothing like conscious experience and thus neither will there be inside a machine. However, the solution might require a conceptual leap.

Consider the possibility that consciousness, albeit physical, is not literally inside the body of the agent—be it biological or artificial. The proposal is that consciousness is the same with the external objects an agent deals with. In this way, the physical properties of the external world might be the same as the properties of conscious experience (Manzotti, 2006, 2017; Manzotti and Chella, 2016).

An example will help. An agent—i.e., a body either biological or a robot—is interacting with an external object, say, a yellow banana. Inside the agent there is nothing with the properties of the banana—being yellow, being elongated, and being slightly bent. When we look for the agent's experience inside the agent's body, we would be compelled to conclude that there is nothing physical with those properties inside the agent's body (yellow, elongated, bent). Not being able to find anything like our experience inside one's body, we may be tempted to conclude that consciousness is indeed particular; that it is invisible, epiphenomenal, not directly measurable, in a world, that it is phenomenal. This option is taken by the hard problem and all its cognate approaches.

We suggest an alternative. When the agent is interacting with the banana, there is a physical entity that is ideally suited to be the same with the agent's experience, namely, the banana itself. The banana is yellow, elongated and slightly bent, just like the experience of it. Nothing else is to be invoked to be the experience of the banana. The banana is better than anything we may ever hope to find inside the agent. The external object scores better than any internal representations.

The advantages of this approach as regards machine consciousness are numerous. There is no need for biological material. There is no need for the emergent property, a very questionable addition to the debate. There is no need to appeal to quantum mechanics, something still alien to the current state of the art in robotics. There is no need to suppose the existence of dubious properties that cannot be observed physically. Everything is measurable, observable and, crucially, causally relevant rather than epiphenomenal. An initial example of this approach, implemented on a robot head, is described in details in Manzotti and Tagliasco (2005).

## Discussion

Four possible objections can be anticipated to this proposal. First objection: the object is not inside the body of the agent, and thus it cannot be either constitutive or the cause of one's experience. This objection has been raised by one of the original proposers of the extended mind, namely by Chalmers (2008). There is no reason to assume that we are located in our head. The physical location of experience cannot be derived from the fact that sensor organs are found on the body. Only the location of sense organs can be estimated by the position of what is perceived. The physical location of consciousness is immaterial, though, as Daniel Dennett's clarified in his famous cautionary tale (Dennett, 1978).

Second objection: the yellow of the banana is not like the yellow of consciousness, or to rephrase it, *the physical yellow is different from the phenomenal yellow*. If we assume that subjective properties are different from physical properties, they could not be the same. This fact, however, is neither self-evident nor empirically found. It is the premise on top of which the hard problem framework got built, an assumption that should be empirically demonstrated rather than assumed. In fact, such a hypothesis is self-confuting—if the two classes of properties were different, we could never see the physical properties. The claim that physical properties are different from subjective properties is unproven. The burden of the proof lies on the shoulder of those who claim there are additional properties. Historically, many scholars argued there where subjective properties because they could not find anything like our experience inside brains. However, external objects are exactly like our experience of them. Therefore, nothing prevents from being the same with our alleged experience of them.

Third objection: the misperception as dreams and hallucinations. Any realist proposal must tackle the issue of misperception. How can the suggested identity between consciousness and external object tackles cases in which the object does not seem to be there? Our reply to such an objection is that the scope of the *present* can be arbitrarily large. Consciousness is made of objects that had causal intercourse with the body of the agent and that, thanks to its neural structure, are still causally active in whatever combinations they happen to be. Consciousness is then always a form of perception, albeit reshuffled and postponed. Of course, this issue alone will require a lot more discussion, but the gist of the strategy is there.

Fourth objection: if consciousness is the same with the external objects, how can the same object look different to different agents? A reply is the following—physical properties are relative, and thus they can be different when compared to a different physical system. The same object can have different physical properties for different agents since different agents have different bodies. The same vehicle can have different velocities relative to different observers moving with as many frames of references. So, the same object can have different properties relative to bodies having different causal properties. The same object will have different colors for tetrachromats, standard trichromats, and color blind of various kinds. Thus, the relative nature of physical properties paves the way to the fact that the same object may indeed have different features for different agents.

## Conclusions

The purpose of this article is to show the problems with GOFAC and thus that it clamors for the adoption of a robust conceptual framework alternative to the Hard Problem and its cognates.

Our proposal offers a new basis for robot consciousness. There will no longer be an elusive property concocted by some particular process inside the body of a robot agent; neither will it be a hard problem. Consciousness is the network of objects and events that, thanks to a body with sensory-motor-cognitive capability are brought to interact together. Consciousness is not an internal property, but the collection of objects that, thanks to the body, are causally responsible for what the body does. The study of robot consciousness will thus shift the focus from internal processes and structures to the analysis of the ontogenetic and epigenetic relations that a body develops and maintains with the external world during its life. Methodologies of developmental robotics (Cangelosi and Schlesinger, 2015) will be a valuable help in this effort.

The presented hypothesis, albeit still in its infancy, offers a complete physicalist alternative—conscious robots would be machines that bring into existence the same relative physical objects human bodies do.

The advent of a conscious robot would eventually lead to new questions about what it means to be a person. The concept of person undergone inclusive variations over the centuries, as discussed by Gunkel (2012). Humanity has come across many problems to include women, slaves and superior mammals in the circle of persons. Today, the problem is two-fold: if we assert that a robot is a kind of person, then the moral responsibility of the robot for its actions must be recognized. On the other side, we have to concede some moral rights to the robot, such as the right of not being switched off.

The concept of person is tightly linked to the concept of consciousness. If an entity can have subjective experiences, and eventually can suffer, then this entity should be treated as a person. In this regard, the studies on robot consciousness may force us to review our fundamental definition of the concept of person.

## Author contributions

All authors listed have made a substantial, direct and intellectual contribution to the work, and approved it for publication.

### Conflict of interest statement

The authors declare that the research was conducted in the absence of any commercial or financial relationships that could be construed as a potential conflict of interest.

## Footnotes

^1^http://www.theswartzfoundation.org/abstracts/2001_summary.asp
